# Toward a New Paradigm in Resistance Training by Means of Velocity Monitoring: A Critical and Challenging Narrative

**DOI:** 10.1186/s40798-022-00513-z

**Published:** 2022-09-16

**Authors:** Juan José González-Badillo, Luis Sánchez-Medina, Juan Ribas-Serna, David Rodríguez-Rosell

**Affiliations:** 1 Physical Performance and Sports Research Center, Universidad Pablo de Olavide, Ctra. de Utrera, km 1, 41013 Seville, Spain; 2 Research, Development and Innovation (R&D+I) Area, Investigation in Medicine and Sport Department, Sevilla Football Club, Seville, Spain; 3 Center for Studies, Research and Sports Medicine, Instituto Navarro del Deporte (IND), Pamplona, Spain; 4 Department of Medical Physiology and Biophysics, University of Seville, Seville, Spain; 5 Department of Sport and Informatics, Universidad Pablo de Olavide, Seville, Spain

**Keywords:** Velocity-based resistance training, Periodization, Training methodology, Level of effort, Training effects, Exercise monitoring

## Abstract

For more than a century, many concepts and several theories and principles pertaining to the goals, organization, methodology and evaluation of the effects of resistance training (RT) have been developed and discussed between coaches and scientists. This cumulative body of knowledge and practices has contributed substantially to the evolution of RT methodology. However, a detailed and rigorous examination of the existing literature reveals many inconsistencies that, unless resolved, could seriously hinder further progress in our field. The purpose of this review is to constructively expose, analyze and discuss a set of anomalies present in the current RT methodology, including: (a) the often inappropriate and misleading terminology used, (b) the need to clarify the aims of RT, (c) the very concept of maximal strength, (d) the control and monitoring of the resistance exercise dose, (e) the existing programming models and (f) the evaluation of training effects. A thorough and unbiased examination of these deficiencies could well lead to the adoption of a revised paradigm for RT. This new paradigm must guarantee a precise knowledge of the loads being applied, the effort they involve and their effects. To the best of our knowledge, currently this can only be achieved by monitoring repetition velocity during training. The main contribution of a velocity-based RT approach is that it provides the necessary information to know the actual training loads that induce a specific effect in each athlete. The correct adoption of this revised paradigm will provide coaches and strength and conditioning professionals with accurate and objective information concerning the applied load (relative load, level of effort and training effect). This knowledge is essential to make rational and informed decisions and to improve the training methodology itself.

## Key Points

- The main limitation of current resistance training practice and methodology fundamentally lies in the necessity to ascertain the degree of effort actually performed by the athlete or practitioner in each training session. The monitoring of repetition velocity contributes decisively to overcoming this limitation.
- A particularly worrying and underlying problem in our field is that of current terminology, which is often inappropriate, misleading and unnecessarily complex, fostering confusion and misconceptions, and hindering the development of a sound and scientific-based training methodology.
- A new paradigm is proposed for resistance training based on the monitoring of movement velocity. This paradigm, supported by rigorous research performed in the last two decades, guarantees precise knowledge of the loads being applied, the effort they involve and their training effects.

## Introduction

As a general concept, a paradigm could be defined as a theoretical framework within which a set of theories are formulated to explain how a certain problem or topic is understood at a particular time. The definition of paradigm that best relates to the so-called sports sciences or exercise sciences is perhaps the one proposed by Kuhn [1] in *The Structure of Scientific Revolutions.* According to this author, the paradigm constitutes the practices and knowledge that define a scientific discipline during a specific period, providing models of problems and solutions to the scientific community. In this context, a paradigm shift involves a major change in the way of understanding and addressing the problems of a discipline that leads to overcoming and ultimately replacing the models and framework prevailing until that time. Thus, a new paradigm arises from detecting an “anomaly” or something that “does not work” or something that does not explain reality or does not explain it sufficiently [1]. Specifically, in the case of resistance training (RT), the “anomaly” or the starting point for adopting a new paradigm lies fundamentally in the necessity to ascertain the degree or level of effort programmed and performed by the athlete or practitioner in each training session. Although there are several key variables that can be manipulated to design and configure RT protocols and programs [2–5], it appears that the level of effort is mainly determined by the relative load (percentage of one-repetition maximum, %1RM) used and the degree of fatigue experienced during each training set [6–11]. Until very recently, our knowledge about these key training variables has been quite deficient, which has prevented knowing with reasonable precision the actual magnitude of load (relative load and degree of fatigue) that resulted in a given training effect. In connection with this fundamental question, a set of “anomalies” or limitations can be outlined that reinforce the need to reconsider this problem. These include, at least, the following: (1) the need to clarify the aims of RT; (2) the very concept of strength, and particularly of “maximal strength,” in the context of physical and sports performance; (3) the prescription of RT (control and monitoring of the exercise dose); (4) the different programming (so-called periodization) models in RT; and (5) the evaluation of training-induced effects. We consider that each of these issues needs to be addressed to clarify common but often misused terms, rationalize decisions, improve RT methodology and direct new research. A thorough and unbiased examination of these core concepts could well lead to the adoption of a revised paradigm for RT.

## The Aims of Resistance Training

RT, also known as “weight training” or “strength training,” typically refers to a form of physical activity that is used to improve neuromuscular fitness and performance by exercising a muscle or muscle group against an external resistance. The health and performance benefits of a properly designed and conducted RT program are large, widely known and well documented [4, 12–15]. In its broadest sense, practically any type of physical exercise could be considered as RT since almost all activities require overcoming some kind of resistance. Nevertheless, in this paper, we will primarily refer to RT as physical exercise performed using external free weights (discs, barbells, dumbbells, etc.) or weight machines that allow for a precise selection of the load to be used in each training exercise. The first question that should be clarified about RT is its very purpose, i.e., what we aim to accomplish when using this type of training. In addition to the resultant physiological effects, expressed, primarily, as structural (peripheral) and neuronal (central) adaptations [16–19], several mechanical effects are generally cited as aims or objectives of RT. These objectives typically include improving the following qualities or manifestations of muscle strength: maximum strength, explosive strength, power, explosive power, rate of force development (RFD), or local muscle endurance [4, 8, 20]. In addition to this large number of objectives, another problem lies in the different types of training protocols proposed to achieve each of them. Thus, RT programs are configured using different relative loads, number of repetitions, movement velocities and exercises [21–26], resulting in different types of RT, such as those typically termed maximum strength training, explosive strength training, ballistic training, power training, RFD training, power–strength training, velocity–strength training, strength–velocity training or muscular endurance training [4, 8, 27]. In our opinion, both the aforementioned training objectives and types of training are misinterpretations of what constitutes RT and how it should be performed. It could even be argued that this plethora of terms fosters confusion since much of this terminology, as we will explain, could be considered inappropriate from a physics standpoint and unhelpful.

From a performance standpoint, there is little doubt that the main aim of RT is to increase the movement velocity developed against any absolute load, and especially against the specific load encountered in competition. This objective is evident when an athlete lifts a particular external load (kg) during a test to assess his or her strength level, but the aim would be the same when the performance in competition depends on moving or displacing the body (or the body together with any external implement) at maximal intended velocity, which typically occurs in actions such as running, cycling, swimming, paddling, jumping, lifting, throwing, hitting, pushing or pulling opponents, and many other specific actions performed in individual or team sports. Thus, the objective here proposed (to be able to move the same absolute loads at increasingly faster velocities) is applicable to any sport except, partially, to weightlifting. In this particular sport, the main goal during competition is to be able to lift increasingly heavier loads at a certain velocity (velocity of the 1RM). However, to achieve this objective (i.e., to lift a heavier maximum absolute load at the same velocity), it is necessary to be able to lift each of the preceding submaximal loads at increasing velocities. Consequently, it seems reasonable to admit that the only possible objective of performance-oriented RT is to increase movement velocity against any absolute load. This, moreover, is a specific measurable outcome to be achieved, which corresponds perfectly with the meaning of the term “objective.” Thereby, if this objective is met, it means that any other secondary mechanical objective of those previously mentioned would have also been accomplished. Indeed, an increase in the velocity developed against a given absolute load for a certain distance means an increment in power output against that load (i.e., the same mechanical work is performed in less time), and it also implies that a greater RFD or “explosive strength” is attained (i.e., greater force applied in less time).

As indicated, just as the main goal of RT is to increase movement velocity against a given absolute load, it could be considered that the only possible type of RT is training for improving maximal strength. This statement is supported by the fact that the only way to increase the movement velocity developed against an absolute load is to apply more force to that load. Thus, in order to achieve the maximum possible velocity against a certain absolute load, the applied force must be the maximum that the subject can exert against that particular load. This statement can be verified by Newton’s second law of physics which is commonly stated in terms of an object’s linear momentum (mass times velocity) following the equation: mass (*m*) times velocity (*v*) equals force (*F*) multiplied by time (*t*) (*m*·*v* = *F*·*t*). If we solve for velocity in this equation, it follows that:

1 $$v=\frac{F\cdot t}{m}$$

Taking into account that velocity equals displacement (*d*) divided by time (*t*), Eq. [2] can be obtained:

2 $$d= \frac{{F\cdot t}^{2}}{m}$$

In equation [1], the mass (*m*) (either that corresponding to a given absolute load, body weight or any sporting implement), and the distance over which the force is applied are stable. Therefore, to increase velocity, it will be necessary to increase the numerator of this equation (*F*·*t*). However, this increase cannot be at the expense of an increase in time, since an increase in velocity for the same distance necessarily implies that the movement takes a shorter time. Similarly, in equation [2], assuming that the mass and displacement (e.g., race distance for a running competition) do not vary, any decrease in time (a key factor to increase velocity), requires an increase in the applied force to maintain equality. Therefore, the only way to increase velocity is to apply more force in less time (i.e., increase the RFD). It thus follows that all types of training that can be performed could well be termed *maximal strength training* (i.e., training for the improvement of maximal strength against the specific competition load), regardless of whether the training effect is assessed by measuring 1RM, maximum isometric strength or by the change in movement velocity against a given submaximal load or set of loads, which are all measures capable of showing the change in the applied force.

**Fig. 1:**
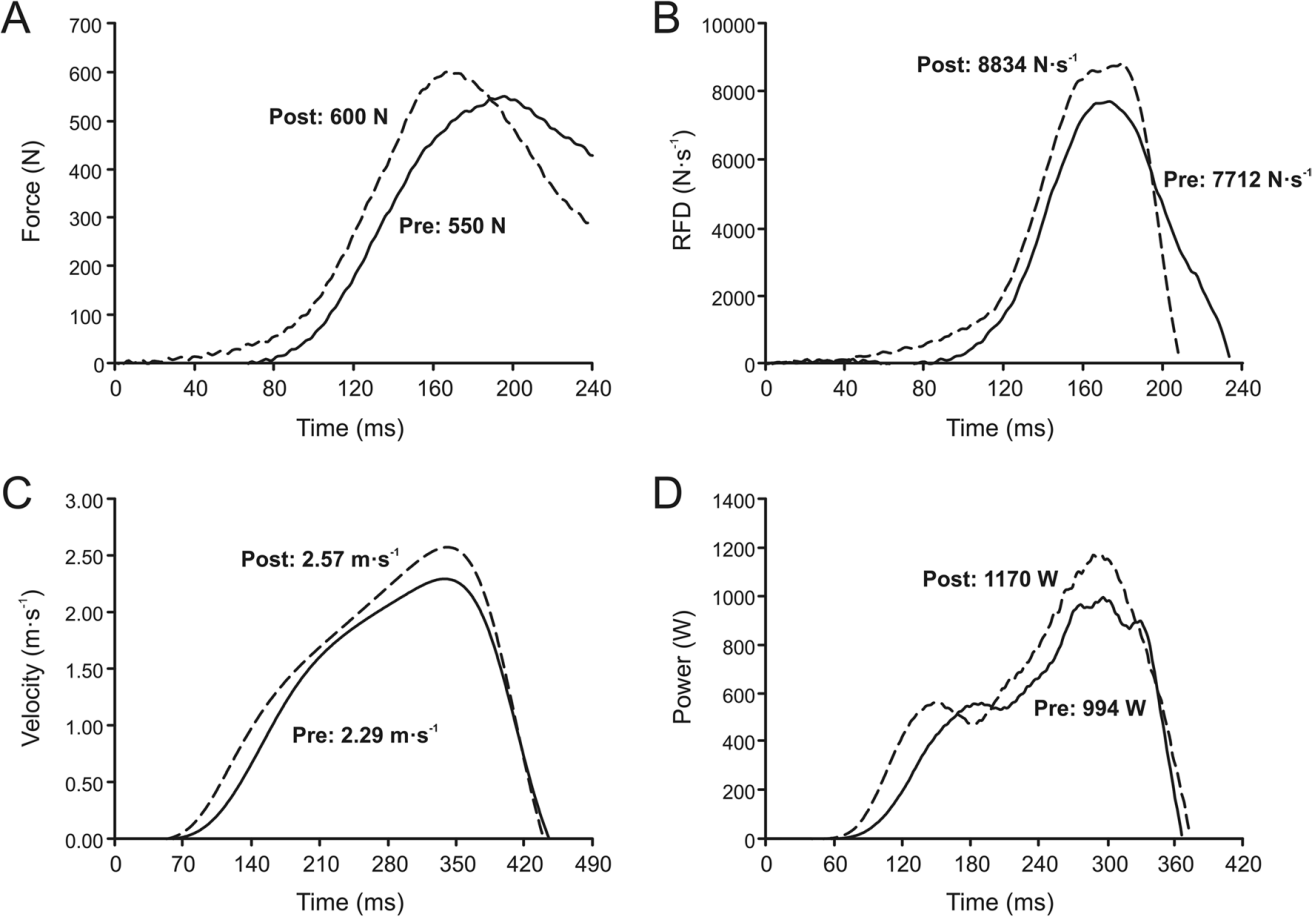
A real example showing changes in selected mechanical variables following an 8-week RT program. Changes from pre- (Pre) to post-training (Post) against a load of 20 kg in the bench press exercise are shown for a representative subject. The force–time (**A**), RFD–time (**B**), velocity–time (**C**) and power–time (**D**) curves were obtained using a force platform synchronized with a linear velocity transducer and sampling vertical force and bar velocity at 1,000 Hz. The *x*-axis values have been adjusted to best show the Pre-Post change in the different mechanical variables. Peak values of each variable are reported

Other types of RT commonly used in the literature (ballistic training, power training, RFD training, and muscular endurance training) could be thought of as different types of training than maximal strength training. However, this is not the case because it is not possible to enhance power output, movement velocity or local muscle endurance without increasing the maximum force applied against the load(s) being used to assess power, velocity, or endurance. Even if the goal were to improve movement velocity or power output against very low loads (as it is usually the case with the so-called ballistic training), this would only be possible if the maximal force applied against these loads increased. Figure 1 provides an example of how it is precisely the changes in the force–time relationship (a steeper force–time curve and a higher and earlier peak force, Fig. 1A, B) following training that allow the development of a faster movement velocity (Fig. 1C) and, as a consequence, an increase in power output against a given absolute load (Fig. 1D). Therefore, since the ultimate goal of RT is always the same, i.e., to improve the maximal force applied against certain absolute load(s), all these other apparently different types of RT are actually maximal strength training (i.e., types of training directed toward the improvement of maximal strength). Thus, in a strict sense, and from a mechanical standpoint, the only possible type of training is maximal strength training; all other types of training do not exist as such.

This concept could be easily explained using the so-called power training as an example. Since power is not an entity on its own but a compound mechanical variable (*F*·*v*), to improve power output, training should be directed toward increasing force application against the load(s) selected. There is no other possible solution to this problem; power cannot be increased unless force increases. Therefore, it could be considered that in reality there is not a specific type of RT that can be termed “power training,” but rather all types of training are, in essence, power training because what is intended and that which precisely allows us to improve power is an increase in the maximal or peak force applied in the corresponding action.

Finally, it could also be considered that one of the aims of RT is to increase muscle hypertrophy. However, for an athlete, a larger cross-sectional area of muscle fibers will only make sense if it allows an increase in the applied force against the same absolute load, which, in turn, will lead to a faster movement velocity. Consequently, in summary, the only possible type of RT that actually exists is maximal strength training, which can be performed at different movement velocities, i.e., using different relative loading intensities (% 1RM). However, in all cases, this will remain maximal strength training.

Based on the above reasoning, the existing terminology related to both the aims or objectives and types of RT could be considered misleading as it often leads to severe misunderstandings. Even though they are widely used and deeply ingrained terms in our field, a close scrutiny of many of these terms reveals that there is an underlying ignorance of the fundamental laws of Newtonian physics. This is a serious problem that hinders the advancement of knowledge in the exercise sciences and, more specifically, in the field of RT. One of the most common mistakes or misconceptions is to believe that power, ballistic, or velocity training are different types of training than maximal strength training, or to consider that the effects of these alleged types of RT occur without improving the maximal (peak) force applied to the loads used to assess performance. Therefore, it seems reasonable to suggest that the concept and denomination of these alleged types of RT should be redefined, if not eliminated altogether. We consider this to be a critical issue that needs to be addressed to improve knowledge about RT and bring terminology in line with science.

## The Concept of Maximal Strength

**Fig. 2:**
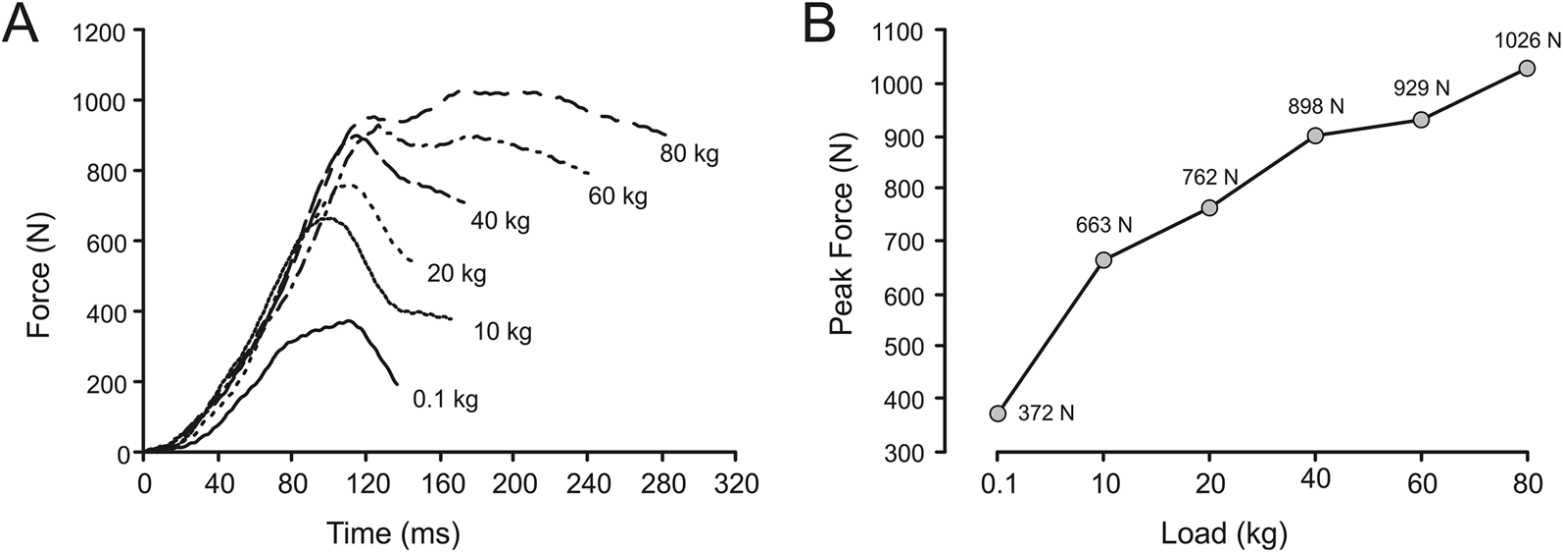
Force application during a progressive loading test in the bench press exercise for a representative subject: **A** force–time curves obtained for each of the increasing loads used; **B** peak force values attained against each load. Data obtained from a force platform sampling vertical force at 1000 Hz

As with the different aims and types of RT, it is also necessary to clarify the concept of maximal strength. Traditionally, maximal strength has been defined as the maximal force that a subject can apply against a load that can only be lifted once over a given distance, or the maximal force applied in an isometric action [4, 28, 29]. Thus, maximal strength is usually determined using a one-repetition maximum (1RM) test or a test of repetitions to failure [4, 6, 8, 29]. This method consists of completing the maximum possible number of repetitions (*n*RM) against a given absolute load to know the approximate relative load (%1RM) corresponding to the load lifted and then estimate the 1RM using a table or formula [3, 4, 6, 8, 11, 29]. However, we find this a reductionist view of the concept of maximal strength because the above definitions represent only one maximum force value: the maximal force developed against the 1RM load, whereas a subject actually possesses as many maximal force values as loads used to be lifted at maximal intended velocity. Therefore, it could be considered that an athlete has infinite values of maximal strength, as many as the loads or resistances to overcome. This issue can be better understood by observing the different values of peak force encountered when lifting different loads during a progressive loading test (Fig. 2), always assuming that maximal intended effort is employed. In this regard, Komi [30] defines strength as “the maximal force or torque a muscle or muscle group can generate at a specified or determined velocity.” Furthermore, Herzog and Ait-Haddou [31] indicate that “the force–velocity relationship defines the maximal force of a muscle as a function of the contraction velocity, measuring the force for the given velocity of shortening at a defined length.” Based on these two definitions, it can be deduced that if the velocities can be different, necessarily, the maximal strength (force) values must also be different. Thus, as indicated above, an athlete does not have only a maximal strength value, but rather he or she will have a maximal strength value for each velocity value reached. Of all these force values, possibly the most important maximal strength value is the one applied by the athlete during the specific competitive action, provided that this action is performed at maximal intended velocity.

**Fig. 3:**
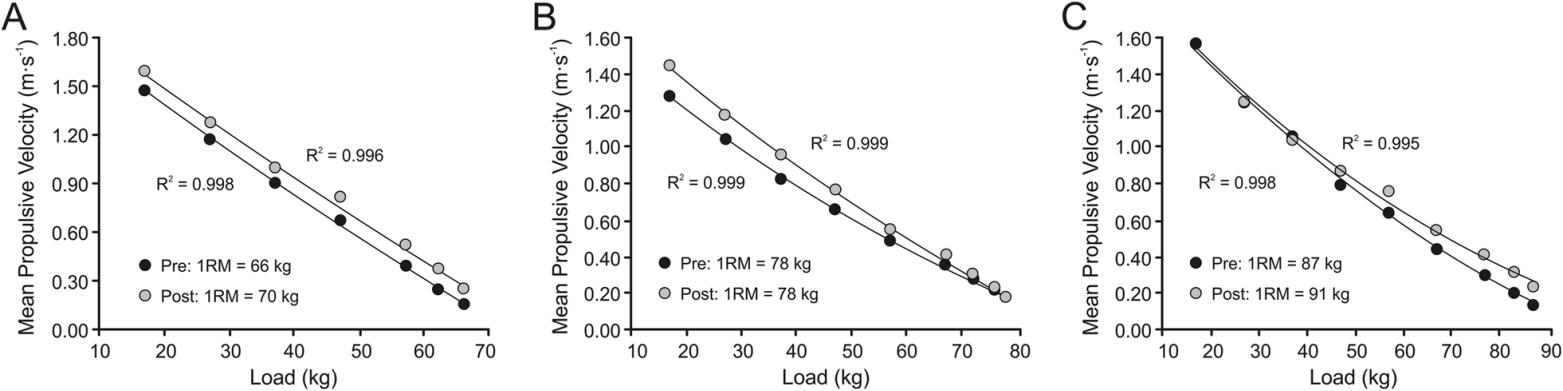
Three real examples of specific changes experienced in the load–velocity relationship following different RT interventions in the bench press exercise. **A** A 6.1% improvement in 1RM is accompanied by a consistent and similar increment in movement velocity against all loads used during the progressive loading test. **B** Despite no change in 1RM from Pre to Post, the subject was able to develop faster movement velocities (which were undoubtedly due to an increased force application) against low-to-moderate loads (20–70 kg). **C** An opposite example, showing a 4.6% improvement in 1RM together with increased velocities against medium and high loads (50–90 kg) but no improvements in the velocity developed against low loads (20–40 kg). Data from absolute loads common to both Pre and Post tests are shown. Velocity data obtained from a linear velocity transducer sampling bar velocity at 1000 Hz

Proper knowledge of the concept of maximal strength allows us to clarify how RT should be performed. It also helps to avoid falling into a set of conceptual and methodological mistakes and misconceptions. Some examples of common inappropriate proposals and assertions could be the following: (1) “we are going to train velocity, but not maximal strength”; (2) “we are going to perform power training, strength–power training, strength-velocity training, velocity–strength training, or strength-explosive training”; (3) “it is necessary to train first strength and then power”; (4) “in many cases, the most important variable is power output rather than strength.” All these assertions are inappropriate because the only possible type of RT is maximal strength training. Therefore, there are no “strength,” “speed,” “power,” or “transfer” exercises, but only exercises performed at different velocities that could improve maximal force application against any absolute load. The absolute velocities used and, particularly, the degree of fatigue incurred during each training set will induce different effects on the force–velocity relationship [23, 24, 32, 33]. Figure 3 shows three real examples of specific changes experienced in the load–velocity relationship following different RT programs. These examples illustrate how, beyond 1RM values, an analysis of the changes induced by different RT interventions on the load–velocity relationship can provide valuable insight into the adaptations brought about by training. In most sports, changes in applied force (and hence velocity) against submaximal loads, far from the maximum (1RM), are much more important for the improvement in athletic performance.

In conclusion, most of the current terminology is unnecessarily and artificially complex which often fosters confusion and misconceptions, hindering the development of a sound scientific-based RT methodology. Practice and research in RT must stem from a rigorous knowledge of the concept of maximal strength, otherwise it is not possible to know why and how strength must be trained.

## The Prescription of Resistance Training: Control and Monitoring of the Exercise Dose

One of the fundamental problems in RT is to ascertain the actual degree or level of effort that each training session entails. In addition to the type, order and number of exercises performed [4, 34, 35], loading intensity and volume are the two main variables determining the effort experienced during RT [8–11]. Intensity during RT is commonly identified with the relative load (% 1RM) associated with the resistance (load) being used or with the magnitude of the absolute load itself (weight), whereas volume is determined from the total number of sets and repetitions per set completed during the training session [3, 4, 6, 8, 11, 36]. Other variables, including training frequency, movement velocity and the duration of the rest pauses between repetitions, sets and exercises also contribute to configuring the resistance exercise stimulus and impact the overall effort undertaken [9, 10, 21, 22, 37–39]. The manipulation of one or more of these variables will modify the training stimuli and potentially elicit different mechanical training effects and neuromuscular adaptations [4, 16, 17, 36]. These effects and adaptations will ultimately manifest themselves in the body’s ability to develop velocity against any absolute load.

Traditionally, the most common indicators used as a reference for dosing the loading intensity during RT have been the 1RM and *n*RM [3, 4, 6, 40, 41]. However, these methods present numerous disadvantages for its use in daily training practice [42–45]. Firstly, as observed in several studies [46–50], the 1RM value changes frequently during the training process. This fact could imply that the actual daily relative training load (% 1RM) may differ from the programmed or intended effort. As a result, coaches will never know the relative load actually used by each athlete in training and, therefore, the real loading magnitude that resulted in a given effect, be it positive or negative. This lack of precise knowledge could lead coaches to assume that the effects obtained from an RT program are due to degrees of effort (training loads) which are different from those actually used during the training intervention [51]. Another major problem is that experience tells us that a high percentage of the 1RMs measured are not true maximums because they are often reached at different (faster) mean velocities than those of each exercise’s own 1RM velocity [42, 52, 53]. Consequently, most of the initial 1RM values established as a reference for programming subsequent training may be wrong. In many training studies, as well as in the everyday practice of RT, it is not uncommon to find that the obtained 1RMs are reached at a faster mean velocity than that of each exercise’s own 1RM velocity (*V*_1RM_) [42], which is comprised within a small velocity range: *V*_1RM_ ≤ 0.20 m s^−1^ for bench press [42, 54], *V*_1RM_ ≤ 0.35 m s^−1^ for squat [52, 55], *V*_1RM_ ≤ 0.50 m s^−1^ for prone bench pull [54, 56], *V*_1RM_ ≤ 0.90 m s^−1^ for the power clean [57], etc. This lack of accuracy in the determination of 1RM results in a 1RM reference value which is lower than the real or true value. This, in turn, means that each of the prescribed loads used during an RT program will constitute a lower relative load than that programmed or intended. In addition, it is important to notice that not all 1RMs are always reached at a very similar velocity in a given exercise for the same subject. In this regard, two or more 1RMs measured with a difference in mean velocity ≥ 0.03 m s^−1^ would no longer be comparable, since a velocity difference of 0.07–0.09 m s^−1^ represents ~ 5% of 1RM (depending on the exercise) [42, 52, 53, 58]. Thus, if this condition is not adequately controlled, the interpretation of the performance changes in maximum strength (1RM) is likely to be incorrect.

On the other hand, the use of the *n*RM method for expressing the relative load is based on the common belief that if several subjects complete the same number of maximum repetitions in a set (each subject using a different absolute load) they are training with the same relative load (% 1RM). However, different studies have shown that not all subjects can perform the same number of repetitions against a certain relative load [7, 59, 60]. In a recent study [61] most of the individuals (56.6% of the sample) performed repetitions above those supposedly achievable (e.g., 12RM, 10RM, 8RM), underestimating the prescribed or intended load in all exercises. It thus seems that the prescription of RT should not be based on a predefined number of repetitions since subjects who exercise using the same maximum number of repetitions per set could be training with quite different relative loads. Moreover, it is very difficult, if not impossible, to know the absolute load with which a subject can perform a given number of maximum repetitions, on a daily basis, for each training exercise. Therefore, using the *n*RM as a reference to gauge the relative load in RT would lead to the same problems already outlined for the 1RM: coaches will never be certain of the relative loads used by athletes in training and hence of the actual exercise intensity that resulted in a given effect.

In addition to relative load, volume is the other key variable that makes up the exercise dose in RT [4, 8]. Although it is common practice for most coaches and strength and conditioning professionals to use a fixed number of repetitions to be completed in each exercise set for all participants, it appears that the level of effort or degree of fatigue depends on the velocity loss incurred in the set or group of sets [62–65] or, more precisely, on the “effort index” [9, 10]. Consequently, if subjects who complete a different number of maximum repetitions against the same relative load (% 1RM) are required to perform a certain, fixed, number of repetitions per set, it is likely that they will experience different degrees of fatigue that will, in turn, elicit distinct training stimuli [7, 59]. This evidence justifies why a prefixed number of repetitions per set should never be programmed, but rather a percent velocity loss to be reached in the set or group of sets should be used, as has been proposed in recent research [23, 32, 50, 65–69].

In brief, the improvement in RT methodology requires a different approach capable of overcoming the aforementioned shortcomings and deficiencies that characterize current methods and practices. This renewed approach must allow for precise knowledge of both the actual load applied and the degree of effort made that results in a given effect, be it either positive or negative. Additionally, coaches should have tools to identify the athletes’ current performance capacity and training possibilities on a daily basis, and this should be achievable without using any invasive or exhausting procedures (e.g., 1RM or *n*RM tests) that could interfere with the scheduled training. To the best of our knowledge, the best way that currently exists to solve these problems resides in the use and monitoring of movement velocity during RT for determining both the relative load used and the degree of effort undertaken [5, 9, 42, 65]. In this regard, very close relationships between movement velocity and relative load (%1RM) have been found for exercises such as the bench press [42, 70–75], prone bench pull [54, 76], squat [52, 55, 72], deadlift [58, 77], pull-up [78, 79], leg press [43] and hip thrust [80], which makes it possible to determine with considerable precision the %1RM that is being used as soon as the first repetition of a set is performed with maximal intended velocity [42]. This is based on the finding that each percentage of the 1RM has its own corresponding mean velocity, and the velocity values associated with each percentage of 1RM have been found to be very stable and reliable, regardless of the subjects' performance level or the change in strength performance after a training period [42, 52, 54, 58, 78]. Therefore, if an athlete lifts a given absolute load with maximal intended effort, the mean velocity reached allows us to estimate with considerable accuracy and certainty the percentage of 1RM that this load represents [5, 42]. Thus, by monitoring movement velocity, it is possible to have objective and real-time information about the relative loads used in each training session and the resulting effects induced throughout the training period. As previously indicated, this latter information comes from the changes observed in the velocity developed against the same absolute load or set of loads: an increase in velocity means improved performance, and vice versa.

Moreover, several studies have shown that the percent velocity loss reached in an exercise set or group of sets is related to the degree of fatigue incurred during RT [5, 9, 10, 62, 64, 65, 81]. Thus, the greater the velocity loss reached in the set against a given relative load, the greater the level of effort [9, 10, 65]. In addition, a very close relationship has been found between the relative loss of repetition velocity in a set and the percentage of performed repetitions [7, 59, 60, 82]. These findings mean that athletes reaching similar percentages of velocity loss in each set (e.g., 30%) against a given relative load in a particular exercise will be experiencing the same or very similar level of effort (e.g., 50% of the maximum possible number of repetitions), although the number of repetitions completed in the set by each athlete may be different [7, 59]. Therefore, the monitoring of movement velocity during RT allows for a precise knowledge of both the main load indicators as well as the resultant training effects, including:

1. The relative load (% 1RM) actually used in each training session.
2. The average mean velocity and degree of effort of each set, session and training cycle.
3. The individual training effect experienced on the entire load–velocity curve after one or several exercise sessions, without the need to perform any 1RM or *n*RM test.

In summary, until very recently, there was no objective method to accurately ascertain the relative load used and degree of fatigue experienced during RT to determine the actual training stimulus that had resulted in a certain effect. Consequently, it seems reasonable to suggest that the conclusions drawn from most studies may be incorrect or flawed, since these are based on false or imprecise indicators of both the load applied and the real effort undertaken [51]. It thus seems timely and necessary to stop taking as a reference variables and methods that have proven to be defective for the prescription and regulation of the RT load.

## Programming Models in Resistance Training

### Periodization Versus Programming

Although widely used, “periodization” is a highly controversial term in the sports science literature. In a strict sense, to periodize means “to establish periods for a historical, cultural or scientific process.” However, this term is commonly used to refer to the temporal organization of the training process, primarily the evolution of relative load and volume during the training cycle [83–85]. The numerous definitions of periodization attest to the confusion surrounding this term [86]. Periodization has been defined as a logical, phasic method of manipulating training variables in order to increase the potential for achieving specific performance goals [87]. In the field of strength and conditioning, periodization postulates the organization of the training program into sequential phases or blocks and cyclical time periods [88]. These phases typically include a “muscular endurance phase,” a “hypertrophy phase,” a “strength phase” and a “power phase” [2–4, 89, 90] which are linked one after another in order to achieve different objectives or specific performance outcomes with the ultimate goal of attempting to reach peak performance at specific times during the season. Proponents of periodization argue that this succession of phases presumably increases the possibility of potentiation in subsequent training phases. However, this, which at first glance may seem like an attractive and interesting strategy, could well turn out to be little more than a nice, well-intentioned, theoretical concept.

The very concept of periodization has been challenged by several authors [86, 91–93] who have expressed serious concerns about the inconsistency of its definition and the lack of sound experimental evidence supporting the concept and its alleged superiority over non-periodized training programs. As pointed out by some of these authors [92, 93], it seems that tradition-based assumptions rather than evidence-based constructs may underlie much of the periodization philosophy.

Moreover, it is particularly striking to observe that the term “periodization” is, in and of itself, often used as a solution to the problems of training, since many authors assert that periodization during RT, per se, allows for the “correct use of loads and recovery times to avoid excessive fatigue and achieve maximum performance at the right, predetermined time” [4, 87, 88, 94, 95]. This has reached the point where if a training program is not labeled as “periodized” it is usually not worth considering. As absurd as it may sound, “periodization” has become some sort of magical word whose use seems to ensure that training is adequate and effective. Nonetheless, the mere act of calling a process, method or strategy “periodization” does not ensure the achievement of any objective.

Contrary to what appears to be suggested, structuring the training process into several periods does not guarantee, per se, that the prescribed training is adequate or that the intended objectives of that training are going to be accomplished. Furthermore, although periodization includes variations in relative load and volume [13, 87, 88, 96], this variation does not guarantee an improvement in physical performance, as the possible alternatives for manipulating these key components of the training load are countless and not all of them ensure a positive effect on performance. Indeed, there is no reason to think that the combination of relative load and volume proposed in a “periodized model” will lead to a performance enhancement for all athletes and situations. Consequently, as it is commonly used in the sports science literature, the term “periodization” does not seem adequate for what it is intended to define and, thus, it would be of no practical value.

Several studies have suggested that a more appropriate term to define the manipulation of the different training variables and to organize the training sessions should be “programming” [50, 87, 97, 98]. Although, as indicated above, establishing certain programming does not guarantee, per se, the achievement of any specific objective, since programming could induce a positive or negative effect, depending on multiple factors. However, the term used is correct because it corresponds to that which is intended: devising and ordering actions. This mainly means “organizing a sequence of efforts to achieve the planned or intended objective,” although the established sequence may not be correct and, in that case, the desired or proposed objectives will not be achieved. It is for all these reasons that, in our view, the term “programming” should preferentially be used instead of “periodization.” In fact, the term periodization is frequently used to express programming [86, 91]: variation of volume, relative loads, and exercises to achieve the best or optimal results at the right time. Yet we face once more the persisting problem of terminology: the existing tendency to introduce unnecessary terms without considering whether they are appropriate and/or necessary to define a certain concept. For example, in the ACSM Position Stand [13] about *Progression Models in Resistance Training for Healthy Adults*, "periodization" is identified with the training principle of “variation,” as follows: “variation*,* or periodization, entails the systematic process of altering one or more program variable(s) over time to allow for the training stimulus to remain challenging and effective.” Other authors have also indicated that periodization introduces “variation” through cyclical phases and time periods [87, 88]. However, as already indicated, "variation" or "altering one or more program variable(s) over time" is nothing more than the act of programming. Therefore, although this process is commonly and inappropriately called “periodization,” the proper term should be “programming” or “organization of training.” Indeed, the main aim of periodization (i.e., to achieve the appropriate adaptation or performance outcomes at a given time) is only a wish, without any sound basis or scientific justification. In any case, a training program, model or strategy will only make sense if it is accompanied by the corresponding manipulation of the variables (conveniently expressed in numbers) that determine the training load. To the best of our knowledge, the best procedure to identify the most adequate or optimal manipulation of the acute RT variables is only through careful experimentation and systematic observation of the relationship existing between the applied loads (stimuli) and the effects (physical and performance changes) produced by those loads. It goes without saying that this problem is not solved simply by giving a certain training program the adjective “periodized.” In our opinion, what is of paramount importance is the manipulation of the numbers that represent the key training variables, such as relative loads and/or lifting velocities, number of sets and repetitions, rest times, exercise frequency, etc., and not the names or terms employed. Put plainly, *training is numbers, not names!* Using the term "periodization" has no meaning or effect on physical performance unless it is accompanied by a detailed numerical expression of the variables that constitute the training load. Unfortunately, this, which should be evident, is often forgotten within a field that considers itself science-based.

The common misuse of the term “periodization” often leads to the self-perpetuating assumption that it guarantees the achievement of the following goals: (a) the appropriate balance between training loads and preparation for competition during the season; (b) fatigue management and reduction in overtraining potential; and (c) adequately staging and timing of performance peaks [87, 88, 99]. Obviously, there is no model, plan, program or training strategy that does not aim to achieve these objectives. Yet it seems unreasonable to accept that all these goals are going to be met simply by giving the model a specific name, in this case "periodized training.” Although it is relevant to identify the main objectives of any training process, the most important issue is to ascertain how to achieve those objectives beyond the introduction of some "appropriate variation" [99, 100]. Much of the literature on periodization seems to embody the underlying belief that almost any form of variation is “good,” yet it is the particular and specific way (through a succession of well-defined efforts) in which that variation in training stimuli takes place that we should focus our attention on.

### The Block Periodization Model

Basically, there are two main periodization models: (1) traditional or parallel models that supposedly consist of the simultaneous development of multiple physical abilities throughout the training process, and (2) block periodization (BP) or sequential models, based on the concept of concentrating the training load into successive “blocks” in order to develop specific physiological systems and motor abilities [87, 88, 90, 101]. Moreover, it has been hypothesized that the concentrated blocks could be structured to allow for multiple peaks within the season, which may be necessary in many modern sports [90, 102]. It is for this reason that BP is often recommended or perceived as a superior model for increasing athletic performance [88, 99, 100, 102, 103].

With regards to RT, BP usually distinguishes between four main phases or objectives: "strength-endurance" or "muscular endurance" phase, "hypertrophy" phase, "maximum strength" phase, and "power" phase [4, 8, 99, 100]. The latter phase is usually divided into a "strength–power" phase and a "power and peak RFD" phase [4, 8, 87, 99, 100]. However, the designation and structure of these phases do not seem to correspond to the observed training effects. This statement is based on the following reasons:

1. In the "strength-endurance" or "muscular endurance" phase, there is also necessarily an effect on muscle hypertrophy, strength and power. In this phase, training usually consists of several sets in which loads of 15-20RM are used in single or multi-joint exercises with 30–60-s rest between sets and exercises [4, 8, 99, 100]. It is reasonable to admit that this type of training is accompanied by gains in muscle mass, especially considering that training with very low loads (30% 1RM) and performing repetitions to failure induces significant hypertrophy [104, 105]. This fact is reinforced when considering that during the subsequent hypertrophy phase the typical recommended training includes repetitions up to 15RM, approximately the same maximum number of repetitions as that proposed for the muscular endurance phase [4, 8]. Furthermore, if this type of training induces muscle hypertrophy, it is very likely that an increase in muscle strength has occurred too, since hypertrophy is one factor that explains muscle strength [16, 106–109]. In addition, training using 8RM, which is typical of the hypertrophy phase, is also considered a specific load for increasing strength [4, 8, 13]. Finally, as indicated in the preceding sections, an increase in maximal strength necessarily implies an enhancement of power output, since an increase in the force applied against a given load leads to a faster movement velocity and, therefore, to the completion of the same mechanical work in less time (i.e., increased power output). Several recent studies reinforce the arguments previously stated, since it has been shown that the changes in muscle endurance capacity depend, at least partially, on increments in maximal strength (1RM). A linear, positive, and significant relationship (r = 0.63–0.71; p < 0.01–0.001) was found between the relative changes in 1RM and the relative changes in the number of repetitions completed against a given absolute load following an RT program [23, 24].
2. In connection with the above, muscular endurance, strength and power should all be improved in the hypertrophy phase. Thus, the hypertrophy phase could also be considered a strength, muscle endurance or power phase. Some questions that could be asked would be: 1) could muscle hypertrophy be increased without an accompanying improvement in muscle strength? and, 2) if the applied force against a given absolute load is increased, is it possible not to improve the power output developed against that load? According to the arguments mentioned above, the answer to both questions is negative.
3. In the third block or phase, the goal is to improve maximum strength. However, it is obvious that if strength has increased, power will also be necessarily improved, and it is likely that hypertrophy and muscle endurance have increased too. As expected, several studies have shown simultaneous changes in these variables following different RT interventions [23, 24, 32, 47, 48, 67] without the need to carry out different training blocks that seek to achieve particular objectives.
4. Finally, as indicated above, it is not possible to increase power output without improving maximal strength, especially when maximal strength is understood in a broad sense and not only as the force developed against maximal or near maximal loads, as previously explained.

One of the arguments usually put forth to support this periodization model is that, in each block or training phase, one single characteristic of physiological development (e.g., endurance, strength, power) is being emphasized [4, 99, 100, 102, 103]. However, this fact is not guaranteed. Indeed, against a given absolute load, the greater the applied force, the greater the improvement in power output. Furthermore, power output may even decrease if the type of training performed during the "power" phase is such that it induces a loss in the maximal force applied against the load(s) used for measuring this variable. Therefore, it appears that the major limitation of traditional periodization (simultaneous development of several physical abilities) does not seem to exist as such since, as it has been argued, it is not possible to achieve some objectives (e.g., increments in muscle endurance, hypertrophy, power or velocity) without achieving another (an improvement in maximal strength). It is a fact that many of these objectives are achieved simultaneously. Therefore, it is proved once again that "naming" the training phases does not contribute to improving our knowledge of the RT process or its methodology. Moreover, it seems clear that the supposed effects to be achieved in each phase are not manifested independently of those that are intended in the other phases.

The sequence of phases, goals and types of training loads is based on the false belief that the effect produced in each phase is required to obtain better results or adaptations in the next block or training phase [4, 13, 99, 102, 103]. According to this, to further improve strength it is necessary to have previously improved or developed muscular endurance and hypertrophy, whereas to improve power it is necessary to have first increased muscle strength [102]. But these deductions or assumptions do not seem to be in line with the results observed in several studies. For example, Mattocks et al. [110] found that hypertrophy-oriented training (4 sets of ~ 8RM-12RM) resulted in a greater change in muscle size than non-hypertrophy-oriented strength training (five 1RM attempts per exercise in each training session), whereas the changes in 1RM strength, isometric and isokinetic peak torque for both the upper- and lower-body were not different between groups. According to these authors, their findings suggest that neither exercise volume nor the change in muscle size from training contributed to greater strength gains compared with just practicing the 1RM test [110]. In addition, this study confirms that hypertrophy-oriented training induces improvements in maximal strength (1RM in this case, as the typical, most widely used, indicator of this effect). Thus, based on these results, it is possible to suggest that: (1) a "hypertrophy phase" is also a "maximal strength phase"; (2) it is not necessary to improve hypertrophy before improving strength; and (3) greater hypertrophy does not necessarily produce greater strength improvements.

Similarly, another study compared the effect of RT programs that employed the same maximum and relative load (%1RM) in each session, but with different training volumes (low vs. high volume) in a group of experienced weightlifters [111]. Although the athletes in the low volume group only performed 65% of the total volume completed by those in the high volume group, no significant differences were observed between groups in strength gains (1RM) measured in the snatch, clean and jerk, and squat exercises [111]. Moreover, several recent studies [23, 24, 32, 48] have shown that, following squat training programs in which the same relative load (% 1RM) was used by all groups in each training session, reaching a velocity loss in each set ≤ 20% (which corresponds to completing less than half of the possible repetitions) tends to induce greater increments in strength, vertical jump and 20 m sprint performance than reaching higher velocity losses (30–45%). However, the groups reaching a higher velocity loss in each set, and therefore a higher level of effort and fatigue, experienced significantly greater increases in muscle hypertrophy compared to the groups experiencing lower velocity losses [32, 48, 112]. On the other hand, Schoenfeld et al. [113] examined the effect of training with different volumes (1, 3 and 5 sets), using the same relative load and number of repetitions per set (8–12RM), on changes in strength and muscle hypertrophy. Results of this study showed that increases in muscle hypertrophy followed a dose–response relationship, with increasingly greater gains obtained with higher training volumes, whereas strength gains were significant and similar for all three groups [113]. Therefore, the results of these studies [23, 24, 32, 48, 110, 113] seem to suggest, again, that it is not necessary to increase muscle hypertrophy before improving strength, and that even greater hypertrophy does not guarantee superior strength gains.

In other regards, it seems unreasonable to indicate that muscle strength should be increased before improving power [8, 99]. As already explained in previous sections, the improvement in the power output developed against a given absolute load is only possible if the "maximal strength" (peak force) applied against that load is increased. Thus, considering that power is the product of force and velocity, the conclusion is the same: if more force is applied against the same load, the result is a faster velocity of that load (the movement is completed in less time for the same distance), and, consequently, an improvement in power output. However, the enhancement of this product (force x velocity) depends exclusively on the increase in force, as the velocity against a given load cannot increase without an increase in the applied force. Greater power cannot be developed unless force application increases since power is a consequence of force application, i.e., it is not possible to improve strength without also improving power. Consequently, the "power phase" and "maximum strength phase" should not be considered as different and independent phases. In addition, as already indicated, the applied training load, particularly the degree of fatigue incurred during the training session, may induce different effects on different zones of the force–velocity curve [23, 24, 32, 33]. However, power output will necessarily be improved against a given absolute load if the applied force increases, regardless of the part of the curve in which the effect occurs. Furthermore, assuming that the effect of training on power is measured by means of actions such as jumps, throws, or movements against light loads, a further performance improvement is likely to occur when RT is performed using low to moderate loads, a low number of repetitions per set and fast lifting velocities (i.e., low degree of fatigue) compared to highly fatiguing training protocols [23, 24, 32, 33, 35, 46, 48, 67, 114, 115]. Consequently, the suggestion or recommendation that it is necessary to previously perform the phases of “muscular endurance,” “hypertrophy” and “maximum strength” to improve power does not seem justified.

### Non-periodized Training

In addition to the problems raised in relation to periodized training, the question becomes more complicated when the term “non-periodized” training (NPT) is introduced. According to the definition of “periodization,” an NPT would be a “training program that does not have any changes (i.e., variation) that would justify differentiating one training period from another.” Therefore, it would be a single period without introducing any type of “variation,” i.e., a training period where the same load, the same stimulus, and the same type of training are applied in every session. However, this situation is unrealistic for at least two reasons: (1) it is unlikely that the same training load can always be applied during successive training sessions for an athlete; and (2) an NPT would not meet one of the basic or core principles of training adaptation, progressive overload [4, 13], which necessarily implies variation in the applied loads [4]. Therefore, it appears that the term “non-periodized” is not particularly useful, although there is a large body of literature devoted to comparing the effect of "periodized" versus "non-periodized" RT [88, 116, 117].

Since in the NPT model variability apparently disappears, it seems that all the advantages of periodized training are lost. Indeed, NPT has traditionally been considered a considerably "less effective" model compared to periodized training [88, 116, 118], precisely because of the lack of variability. However, NPT could have many configuration alternatives that introduce some variability elements, which inevitably translate into actual changes in the training load and, thus, entail a breach of the NPT program. According to several authors, the periodized models involve continuous changes in relative load and volume, with a tendency to increase the relative load and decrease the volume over the training period [87, 88, 99, 119, 120]. Therefore, if the relative load remains stable throughout a training cycle, training could be considered “non-periodized.” For example, during NPT, performing repetitions "up to muscular failure" is typically used [87, 88, 116, 118]. In this case, athletes perform the same number of sets and repetitions per set (e.g., 3 × 8RM) in all sessions of the training cycle [117, 121–123]. However, although it appears that RT using repetitions to failure does not produce the greatest performance gains [23, 24, 32, 48, 124, 125], this type of training is very likely to result in strength gains [126, 127]. Therefore, if we aim to maintain the same number of maximum repetitions in each set (e.g., 8RM, 6RM or 4RM) for a given exercise during the training period, it would be necessary to modify (increase) the absolute load used in training. But if the absolute load is changed, it could be admitted that there has been some "variability" in the actual training load, which is characteristic of periodized training. Therefore, what happens if the relative load is maintained but the absolute load increases? Can this be considered as a change in training load (variability)? And, consequently, is this periodized training, or is it not? We think that this change in the training load could be considered as a variation, which is related to training periodization. In addition, although the relative load is maintained, an increase in the absolute load is an irrefutable proof that there has been an improvement in performance in the trained exercise*.* This situation would indicate that the NPT program has induced a positive effect. Therefore, a true or “pure” NPT is not possible in practice and, in some cases, maintaining the relative loading intensity throughout a training cycle, or even tending to reduce it, may be the best indicator of a positive training effect [51].

Finally, since NPT consists of maintaining a fixed and predetermined maximum number of repetitions during the training period, this type of training presents an additional problem. If all subjects train with an individually determined absolute load (kg) that allows them to perform the same number of repetitions (e.g., 8RM), it is highly unlikely that they are training with the same relative load (%1RM). This results from the fact that the number of maximum repetitions that can be completed against a certain relative load (%1RM) presents a high inter-subject variability [7, 59, 61]. This problem is not unique to the NPT model, but it is also applicable to any other periodized training model in which the load is applied and quantified using the *n*RM method.

### The Structure of Periodized Training Cycles: Macrocycle, Mesocycle and Microcycle

Different authors have established a "hierarchical periodized system" with different levels (time periods and fitness phases) for the organization of training [88–90, 99, 100, 102, 103, 119, 120, 128]. Although the terms used to categorize the types and methods of training and the proposed objectives for the different time periods may slightly differ among authors, an annual training plan is usually organized into distinct cycles: the macrocycle, the mesocycle, the microcycle, and the training session [88, 100, 128]. The macrocycle is typically defined as a large duration cycle lasting approximately 6–12 months. Traditionally, it is divided into three main periods: preparatory, competitive and transitional, which are further divided into a system of mesocycles and microcycles. The mesocycle is a middle-length cycle consisting of several microcycles, with a usual duration of 3–4 weeks, although it can reach up to 12 weeks. Finally, the microcycle is identified as a small training cycle (typically 1–2 weeks, but even up to 4 weeks) with a different number of training sessions. As a justification for this hierarchical structure, it has been proposed that these time periods or phases are necessary to carry out a set of actions and activities that lead to the fulfillment of different specific training objectives. However, it turns out that the training effects and the times required for such effects to occur are not determined by the wishes of the programmer. In response to a theoretically similar training load, the effects and times of adaptation can vary considerably among different subjects, according to each subject's characteristics and even to the particular situation of the same subject at different times of the season. In this regard, it would be possible to differentiate between distinct periods or phases depending on the type of training that is carried out, but this distinction should not be based on the effect that may be produced, because this effect cannot be guaranteed, nor can it be verified in most cases. In addition, as can be observed, there is a great disparity in the duration proposed for each of these levels of structuring. The absence of consensus on the specific duration for each of these hierarchical levels and the lack of agreement between what is intended to achieve and what can be obtained in each of these periods make the use of this terminology somewhat confusing, useless and, above all, unnecessary. In our opinion, the use of the term cycle (or phase or period) adding its duration in units of time (days, weeks or months) is simpler and more understandable.

### An Alternative Block Periodization Approach: Mesocycles of Accumulation, Transmutation and Realization

Another prevalent block periodization is known as the “multi-targeted BP training system” [90, 102, 128]. The main structural unit of this training model consists of training blocks lasting 2–4 weeks called “mesocycles,” and each block or mesocycle includes highly concentrated workloads directed at a minimal number of training modalities [90, 102, 128]. The objectives are aimed at the consecutive development of specific skills that, according to the authors, contribute to an “optimal interaction and overlap between them” [128]. This approach is very similar to the “single-targeted BP” previously described, and its justification is practically the same: overcoming the limitations of the traditional (i.e., parallel) periodization model [87, 88]. The main differences between the “single-targeted BP” and “multi-targeted BP” training systems are as follows: (1) The name of each block. Whereas in the “single-targeted BP”, training is usually structured in four blocks termed "phases": muscle endurance, hypertrophy, maximal strength and power or RFD phases [87, 88], “multi-targeted BP” only contains three blocks: accumulation, transmutation, and realization [90, 102, 128]; (2) training orientation. The “single-targeted BP” is mainly focused on the organization of RT, while the “multi-targeted BP” refers to RT and training of other qualities such as endurance, aerobic and anaerobic power, and sporting ability; and (3) the duration of the training period. Usually, the “multi-targeted BP” is shorter than the “single-targeted BP.” However, it appears that the proposal for the evolution of the training load is virtually the same in both periodization models [129], although this is just a presumption, since nothing is specified on specific volumes, relative loads or types of exercises to use in the description of the phases and mesocycles; only a short overview in qualitative terms is provided [87, 88, 90, 102, 128]. In this regard, the “accumulation” mesocycle is characterized by relatively high volume and medium intensity workloads [90, 128]. When applied to RT, this type of training could be identified with the “muscular endurance” and “hypertrophy” phases of BP [87–89]. The “transmutation” mesocycle is characterized by a higher relative load and a reduced training volume, which could be equivalent to the “strength” phase in the single-targeted BP. The same parallelism is observed for the “realization” mesocycle, in which the training load is supposedly more specific with the aim of improving “muscle power.” In short, it appears that dividing the training cycle into three phases or blocks and giving them a new name (accumulation, transmutation, and realization) is sufficient to achieve the intended objectives and an optimal interaction and complementation between them. However, it seems evident that simply renaming a training phase, block or period does not mean that an adequate, performance-enhancing, training is being programmed and carried out. To our knowledge, the effectiveness of a training model will be determined by the type, magnitude and duration of the prescribed training load in relation to the needs of each athlete [4, 17, 36], not by the name(s) given to the training model or approach.

In addition, and in relation to the above, another aspect worth considering is the terminology used in this alternative BP approach. A careful and thorough examination of the terms used reveals serious inconsistencies. Firstly, the term “accumulation” refers to “gathering a large number of things or collecting a remarkable amount of something.” The main issue here lies in the very meaning of the term “accumulation.” In this regard, several interesting questions could be asked: what is accumulated? how does it accumulate? and, when the accumulation block ends, does this mean that nothing else is “accumulated” from that point on? Moreover, if the “accumulation” block consists of physiological, biochemical, physical and technical skill changes for inducing adaptations in general aerobic endurance, muscle strength and basic technique [90, 102], what happens during the rest of the training cycle? Is nothing “accumulated” anymore? This proposal does not seem to make sense, since it is not in accordance with the process of continuous biological adaptation that occurs during training. On the other hand, this term is used and justified because this "accumulation" is supposed to serve as a basis for subsequent specific preparation [90, 102, 128]. It is probably true that during a training cycle or program it is necessary to apply different types of loads at different times to comply with the basic principles of training: overload, progression, variability, and specificity [4]. However, it seems unreasonable to think that during some training periods or mesocycles something “accumulates,” whereas in others it does not. Therefore, we can consider that this terminology does not contribute anything to the improvement in training methodology. Moreover, it seems erroneous to call a mesocycle “accumulation” because the physiological processes that take place in response to training do not correspond to the meaning of the term. Even if the term “accumulation” were accepted, could this "accumulation" be attributed only to a certain block or mesocycle considering that all training sessions performed in said block aimed to "accumulate" something? In our view, the term “accumulation” is inappropriate to describe or refer to biological processes. In addition, it cannot be justified that, by mere decision of the "programmer" (coach, trainer or strength and conditioning professional), nothing else is going to be accumulated from a certain day onwards. This would be equivalent to ordering that no training effect should occur from a certain moment.

The following block is called “transmutation” or “transformation,” which means to change or turn something into something else. Based on this concept, it is proposed that the motor and technical capacities, previously accumulated in the “accumulation” block, are transformed so that they are now transferred to specific performance capacities [90, 102, 103, 128]. To our knowledge, there is no known physiological process to explain that an attribute, characteristic or capacity can “change form” or “become something else.” Additionally, it does not seem appropriate to indicate that there should exist a transmutation or transformation phase within a training cycle because physiological changes or "transformations" do occur continuously throughout the training cycle. For example, it is usual to hear from coaches (and even to find in the literature) that a training mesocycle was programmed to transform maximal strength into rapid strength or RFD, wrongly called “explosive strength” [130, 131]. However, strength cannot be "transformed" into anything. In any case, the only thing that could be “transformed” (i.e., change in shape) is the force–time or force–velocity curve. Yet these curves do actually change, or at least that is what should be intended, from the very first training session to the last one of a training cycle and, ultimately, throughout an athlete’s entire sporting life. Changes in these curves will occur as a consequence of training-induced structural, neural, and functional adaptations [16, 17, 36]. Therefore, if during the previous “accumulation” block no transformations in the force–time or force–velocity curves (which entail changes in the neuromuscular system at the physiological level) were produced, what is the use of training? And if, as expected, transformations did occur during the “accumulation” block, that block would also be a “transformation” mesocycle. Thus, once again, the term "transmutation" or "transformation" does not provide any valuable knowledge or insight into the programming or methodology of RT.

Finally, the last block is called "realization" and lasts about 8–15 days [90, 103, 128]. According to different authors [87, 88, 90, 103, 128], this mesocycle was designed as a pre-competitive training phase and its main aim is to restore the athletes and prepare them for the forthcoming competition using drills for modeling competitive performance and a sport-specific program for quick active recovery. Thus, during this final block, the motor and technical capacities “accumulated” and “transformed” in the previous mesocycles are used to obtain the best possible competitive results [90, 103, 128]. In other words, it is assumed that the athletes will obtain a great benefit or improvement in their sporting performance because the training “accumulated” and “transformed” in the previous mesocycles has prepared them for obtaining an increased specific performance after applying a final “realization” block. However, the term “realization” means “to carry out something or perform an action.” It is a very common term that does not allow us to express, per se, any distinctive feature of this block with respect to other moments of the training cycle. It is important to consider that, at any time during the training cycle or session, some activity or action is being “realized.” Therefore, it seems clear that the term “realization” is inaccurate and inadequate for denoting biological processes, adaptations, types of training, or effects on performance.

In brief, a training program including the succession of the so-called accumulation, transformation and realization mesocycles is not a guarantee of success. Any coach or strength and conditioning specialist could design an effective or inappropriate training program, regardless of whether or not they use this convoluted terminology. This is so because the terminology does not determine the program's quality. It would be a serious mistake to think that if coaches use these terms to define and design RT programs, the prescribed training is going to be adequate or optimal for inducing the desired neuromuscular adaptations. Even when considering that the terminology is not the most important aspect of RT methodology, it is necessary to understand and highlight that the use of inadequate terms does not contribute anything positive to the development of the science of sports training. On the contrary, this unfortunate terminology constitutes a grievous impediment to advancement in our field, often leading to confusion and making it very difficult to ascertain the actual training that has been carried out. In our opinion, this artificially sophisticated and unnecessarily complex terminology is empty of content. Use of the “accumulation,” “transmutation,” and “realization” terms seems to suggest or guarantee that training is well designed which prevents an adequate reflection on relevant aspects (variations in relative loads, volume, frequency, exercises, etc.) of the RT design, thus reducing the possibilities of improving the training methodology. The real problem lies in converting these names into numbers, magnitudes of training loads adjusted to the needs of each sport, capacity to be developed, and particular athlete. If the application of the successive training loads is correct, and it is carried out during a period adjusted to the adaptation time of such training loads, a positive effect on performance is very likely, regardless of the names (or absence thereof) given to the training process or its phases.

## Alternative to Traditional and Block Periodization Models

### Potential Interference Effects and Type and Magnitude of the Training Loads

As already mentioned, the traditional periodization model proposes a sequencing of different and numerous targets to be simultaneously developed (from general to specific contents and from lower to higher exercise intensities) during the preparatory and competitive periods [90, 102]. In addition to other factors, this aspect is postulated as the major limitation of traditional periodization [90, 128] for the training of high-performance athletes in endurance, combat sports, ball games and aesthetic sports. The block models (both single-targeted BP and multi-targeted BP) reduce the number and duration of the phases and goals in each phase [88, 90, 102, 128]. However, in general, the proposal remains similar to that of the traditional model in terms of RT, since in these models a great emphasis is placed on the sequencing of objectives to avoid simultaneity and incompatibility between them. Nevertheless, as already discussed, we can consider that there is a single fundamental objective for RT: to increase the maximal force applied to any load and in any physical–technical action, be it general or specific. If this objective is met, any other goal will be simultaneously achieved, including the technical improvements, since it is not possible to improve technical execution without increasing the applied force in the technical action when the goal is to achieve the maximum execution velocity. In addition, it has been shown that it is possible to achieve this objective without interfering with other components of physical performance, provided that the RT applied induces a low level of fatigue [51, 132–137].

We think it is important to point out that the incompatibility (potential interference effects) of RT with the development of other components of physical fitness does not lie in the fact that several objectives are attempted simultaneously, but rather in the type and magnitude of the loads applied during the training program. Based on much of the existing literature [4, 6, 8, 13], it appears that the main and practically the only RT method used to improve maximal strength is to perform the exercise training sets to muscle failure. However, there is a growing body of compelling evidence indicating that it is unnecessary to complete the maximal number of repetitions in each training set in order to obtain very significant improvements in maximal strength [23, 24, 32, 46–48, 50, 111, 124, 125, 138]. On the contrary, it appears that completing sets to failure reduces the potential strength gains compared to performing fewer repetitions per set against the same relative loads [23, 24, 48]. Training programs in which the same range of relative loads (% 1RM) were used resulted in quite different effects on high-velocity performance actions (i.e., sport-related activities) depending on the degree of fatigue (as reflected and quantified by the percent loss of repetition velocity in each set) experienced [23, 24, 32, 46–48]. For example, recent studies [23, 32] have found that squat training against loads of 70–85% 1RM was less effective in inducing strength gains (1RM: + 13.5%) and vertical jump performance (CMJ: + 3.7%), and even resulted in detrimental effects on 20 m sprint running (T20: -1.0%), when 40–45% velocity loss (VL) was reached in each set compared to 20% VL (1RM: + 17.6%; CMJ: + 9.1%; T20: + 0.3%) or 10% VL (1RM: + 17.9%; CMJ: + 9.2%; T20: + 1.5%). To reach 40–45% VL in the squat exercise means to exercise close to muscle failure in most sets [59], whereas a VL of 20% or 10% implies performing approximately 50% and 30%, respectively, of the possible repetitions in each set against these loads [59]. Similarly, in a subsequent study [24], using the same RT methodology but lower loads (55–70% 1RM), comparable results were obtained: a 45% VL (close to failure) resulted in significant improvements in 1RM (+ 15%) and CMJ (+ 4.9%), and a slight, nonsignificant, improvement in T20 (+ 0.5%), whereas a 10% VL induced significant gains in 1RM (+ 22.5%), CMJ (+ 11.9%) and T20 performance (+ 2.4%) [24]. It is important to note that, in all these studies [23, 24, 32], only the squat exercise was used; vertical jumps and running sprints were not included as training exercises during the intervention period. Based on these findings, it appears that the degree or potential of transference from squat training to these high-speed sports performance actions was not only determined by the relative loads used but, especially, by the actual degree of fatigue incurred in each set [23, 24]. Taken together, these results reinforce our contention that the incompatibility does not lie in the “ability” of RT programs to induce improvements in maximal strength and high-speed actions concurrently, but in the type and magnitude of the loads applied during the training program. Thus, as long as the correct relative loads are selected and the level of effort is far from maximal, which implies a low loss of repetition velocity in each set, RT is most likely to result in positive performance adaptations and minimization of potential interferences.

### Programming Models and Sequencing of Training Loads

All the models previously discussed are based on the need to comply with an order or sequence in the application of training loads. This procedure supposedly allows athletes to: (1) obtain more relevant performance improvements, and (2) achieve different objectives. However, the main problems pertaining to RT do not seem to be the training models or the sequencing of loads because, as previously discussed, the goals established for each phase are necessarily achieved in other phases as well, and, moreover, it seems that it is not necessary to achieve a given objective in a certain phase to attain another objective in the following phase(s). To our knowledge, the fundamental problem that needs to be addressed in RT is to improve our knowledge of the following key variables:

1. The relative load (%1RM) associated with the absolute loads used in each training session. This should be accomplished in real time and without using invasive procedures that may interfere with the training process.
2. The actual level of effort or fatigue experienced during each exercise set and session.
3. The effects that are taking place, on a daily basis, throughout the training cycle. This information should be obtained without using any specific test or procedure different from the actions being performed during the training process.

Obtaining accurate and objective information pertaining to these three variables would be of great value to make rational and informed decisions for an adequate configuration of the resistance exercise protocols and the organization and design of RT. These decisions will be rational and informed because they will be based on a more precise knowledge of the training loads being applied (relative loads and level of fatigue) and the effect that these loads are producing. Obviously, knowledge of this information does not prevent coaches and strength and conditioning professionals from making mistakes in the appropriate choice of the training loads applied in each session. However, unlike what has happened to date, coaches will have accurate information about what athletes are actually doing, what load has been applied (something not satisfactorily known to date), and, consequently, how to make decisions to improve RT. Based on the information provided by these variables, the prescription of training could be improved and regulation of the training loads will depend on: (1) the daily physical condition of the athlete, and (2) the effect that the applied loads are producing.

In connection with the above, it should be highlighted that no specific model or sequence of training loads (especially when these are expressed in relative terms, %1RM) could be proposed as the best model or the optimal succession of loads to achieve a particular goal. The mechano-chemical transduction response to a given training load is different for each individual. This response fundamentally depends on genetic endowment, although several other factors including training background, age or sex also seem to play a role [36, 139, 140]. Thus, the training loads applied during each training session and cycle should be adapted to the particular characteristics and needs of each athlete (sports modality, current physical development, performance status, phase of sporting life, etc.) to promote the desired adaptations. To achieve this goal, it is essential to carry out a rational sequencing of the loads applied based on the knowledge of the three above-mentioned variables: relative load, level of effort and training effect.

Knowledge of these three variables has allowed us to verify experimentally that, in some cases, a stable or even regressive sequencing of relative loads during the training cycle (with a level of effort far from failure) is enough to improve maximal strength [51, 97, 141]. The only condition is that there must be a tendency to increase the absolute load throughout the cycle. Thus, for a given increase in absolute load, a greater improvement observed in maximum strength will mean a greater decrease in the relative load used during the training cycle. Therefore, based on these findings and deductions, it appears that the best situation that can occur when applying an RT program is one in which the athlete's response allows a gradual increase in the absolute load, but not in the relative load. Interestingly, this would correspond to a “non-periodized” training program. This situation would be the most positive scenario for athletes and coaches, as it means a progression in performance without noticeable fatigue and a low risk of injury [5, 51, 65]. It is clear that this proposal is far from the common position stands of many exercise associations which recommend a necessary, if not mandatory, progression of relative loads during the training cycle and always performing repetitions to muscle failure in each training set for improving maximal strength [4, 8, 13]. The here presented proposal is not intended to establish a new model of RT programming, but rather to highlight the importance of carrying out rational and practical actions to improve the RT methodology and maximize strength performance, and for this, it is necessary to rely on the information provided by the three variables indicated above.

In summary, in this section it has been shown that there is not an optimal or best RT model or progression of loads for improving maximal strength. In any case, what could and should exist is a rational application of the principles of overload, progression, and individualization based on each athlete's response to the loads being applied and the observed training effects. Furthermore, experience and experimentation seem to confirm that, in many cases, a load progression in absolute terms, with a slight or no increase (sometimes even a decrease!) in the progression of the relative load during the training cycle could be enough for inducing significant strength improvements [51, 141]. As further strength development occurs, it is likely that both relative and absolute loads will have to be increased throughout the cycle, although this situation will be determined mainly by the specific response of each athlete to the applied training loads.

## Velocity Monitoring as a Fundamental Contribution to the Regulation of the Resistance Training Load

The finding that mean velocity could be used as a very good estimate of the relative load being lifted was first published in English in 2010 [42], but its origins date back to pioneering work by González-Badillo in the 1980s and 1990s [142–144]. The evidence that repetition velocity could constitute the best reference to gauge the real effort experienced by the athlete certainly represented a breakthrough in the field of RT, with many key implications and applications derived from it. Numerous studies carried out in the last decade have shown that the contributions of monitoring movement velocity for the improvement of RT methodology are numerous and of great practical importance [5, 7, 9, 10, 22, 32, 42, 51, 52, 54, 55, 59, 63, 65, 67–69, 78, 81, 145–148]. It is pertinent to note that when using movement velocity for the regulation of the training load, as well as for assessing the training effect, it is necessary to comply with an essential requirement: each repetition must be lifted at maximal intended or voluntary velocity [5, 42, 52, 65]. This condition, in addition to being necessary for monitoring the main RT variables (i.e., relative load and training volume), has been shown to induce greater strength gains and improvements in jump and sprint performance than performing repetitions deliberately more slowly [21, 22, 149, 150]. The main contributions of the use of movement velocity to improve the RT methodology are next briefly described.

### Relative Load

Monitoring movement velocity allows us to determine the relative load (%1RM) that is being used as soon as the first (fastest) repetition against a given absolute load (kg) is performed at maximal intended velocity [5, 42, 52, 54, 58, 76–78]. This is based on the finding that each percentage of the 1RM has its own mean velocity [5, 42, 52, 54]. If bar velocity is monitored with the appropriate technology, this key information would be immediately and readily available (i.e., in real time), which constitutes an important advantage for a better regulation of the athlete's effort and the monitoring of the training load. In addition, the velocity values corresponding to each %1RM are specific to each exercise and very stable, regardless of the subjects' performance level or the change in strength performance that occurred following a training period [5, 42, 52, 54, 78]. This important finding allows us to ascertain the degree of effort associated with the first repetition (usually the fastest one) of an exercise set as soon as this repetition is performed. Thus, the slower the movement velocity, the greater the degree of effort, as the absolute load lifted will represent a higher %1RM [5, 42].

### Velocity Loss

In addition to the first repetition’s mean velocity (which indicates the %1RM being used in a given exercise), the loss of repetition velocity in each exercise set turns out to be a very precise indicator of the actual degree of effort or fatigue experienced as successive repetitions within a set are performed against a given relative load [5, 7, 9, 10, 59, 65]. Recent studies have found a very close relationship between the relative loss of velocity in each set and the percentage of performed repetitions against different relative loads in several exercises [5, 7, 59, 60, 78]. These results suggest that, for a given relative load, subjects who reach the same percentage of velocity loss in each exercise set will be experiencing a very similar degree of effort or fatigue, although each subject might need to perform a different number of repetitions per set [7, 59].

### Degree of Effort in Each Set or Group of Sets: The “Effort Index”

The information provided by these two key variables (first repetition’s velocity and velocity loss in each set) is complementary, and it allows us to estimate the overall effort experienced by each athlete in the exercise sets that make up each session and training cycle. The product of both variables provides a single indicator of effort or fatigue that we have termed the “effort index” (EI) [9, 10]. The validity of this novel index as an indicator of the degree of fatigue has been confirmed by the close relationship observed between the EI and several physiological and mechanical markers of fatigue [65, 151–153]. Positive and significant correlations have been found between the EI and the loss of velocity against the individual load that elicited a 1 m s^−1^ pre-exercise velocity (*r* = 0.92 and *r* = 0.98 for the full squat and bench press, respectively; *p* < 0.001), and the loss of CMJ height pre-post exercise (*r* = 0.93; *p* < 0.001) [9, 10]. Furthermore, the EI also seems a good predictor of metabolic stress based on the strong correlations found with post-exercise lactate concentrations (*r* = 0.90 and r = 0.98 for the full squat and bench press exercises, respectively; *p* < 0.001) [9, 10].

### Training Effect in Each Session

By monitoring repetition velocity, it is possible to quantify the training effect occurring in each training session. For this purpose, it is enough to compare the velocity attained against the same absolute loads during successive training sessions. Thus, if a certain absolute load is lifted at a faster mean velocity than in the previous sessions, it means that this load has come to represent a lower %1RM and, therefore, that the maximal force applied to that load and, very likely, the corresponding 1RM value would have improved. In contrast, if the velocity attained against the same absolute load decreases in successive training sessions, the training effect would be negative. This procedure allows us to assess the training effect in each session with high precision and without the need to perform any 1RM or *n*RM tests [42, 52, 54]. As an example, let us suppose that an athlete lifts a given absolute load at a 1 m s^−1^ mean propulsive velocity in the full squat exercise, which represents approximately 60% 1RM [52]. If, following several training sessions, the same absolute load is lifted at a faster velocity (e.g., 1.07 m s^−1^), we could be almost certain that the maximum strength of the athlete would have improved, and that increment can be quantified. In this example, the load would now represent ~ 55% 1RM since the velocity difference between each 5% 1RM in this exercise (full squat) corresponds to ~ 0.07 to 0.08 m s^−1^ [52], so that the athlete would have improved his or her strength performance by ~ 5%.

In brief, with the appropriate monitoring of the above-mentioned three variables (relative load, level of effort and training effect), it is possible to rationally and systematically apply the principles of overload, progression, and individualization of the training load, without the need to perform any demanding, time-consuming and interfering 1RM or *n*RM tests to know the strength level of the athletes. Therefore, it appears that there is not a specific and necessary model (characterized by certain relative loads, progression or sequencing of loads, number of sets and repetitions per set, level of effort, particular objectives, etc.) that must be followed or applied to improve strength performance. On the contrary, through the proper use of these three variables, it is possible to obtain the most accurate information about the load applied and the training effect. Precise knowledge of the load actually applied and its resulting effect is of paramount importance to establish the cause–effect relationships that should be at the basis of rational decision-making and the improvement in RT methodology. In addition, the monitoring and management of the relative loads used and the level of effort experienced in each training session are critical for a better understanding of the physiological factors underlying performance changes [9, 10, 62, 64, 65]. A highly accurate recording of relative load, level of effort or fatigue, and training effect for each training session could be considered as the best criterion for the development of any regression equation aimed at validating any structural, chemical, molecular, or neural factor as a precursor of training effects.

## Concerning the Effect of Resistance Training Programs

Part of this topic has been addressed in previous sections, although we consider it necessary to highlight some relevant issues. Traditionally, muscle strength has been assessed by performing 1RM or *n*RM tests [4, 8, 13, 29]. Less frequently, maximal isometric or isokinetic strength tests have also been employed [28, 154, 155]. As already explained, to overcome the shortcomings and limitations of using 1RM or *n*RM tests [42, 52], the most accurate and objective procedure is to quantify the changes in movement velocity at the end of the training cycle (post-training) against the same absolute loads used in the initial or pre-training test [23, 24, 51, 133]. This method is the same as the one described for the assessment of the training effect in each session but uses a greater number of loads and takes as a reference the absolute loads measured in the initial test. The application of this procedure presents two significant advantages: (1) the loads used to assess the training effect are the same pre- and post-training; and (2) the change in movement velocity against each absolute load is the most accurate and useful indicator for assessing changes in the applied force: for the same mass (kg) and distance of force application, the observed change in velocity is directly proportional to the change in applied force. Thus, according to the change in movement velocity, the change in force could be estimated. For example, in the squat [52] and bench press exercises [42, 54], a change in movement velocity of 0.07–0.08 m s^−1^ against a certain absolute load is equivalent to a ~ 5% change in 1RM. However, this calculation is not even necessary, as the change in movement velocity against a certain submaximal absolute load is what determines athletic or sporting performance in most cases. Indeed, the main performance goal in many sports competitions is to move the same absolute load (body weight, or body weight plus an additional implement) at a faster velocity. This increase in movement velocity is critical for many individual sports and many specific actions performed in team sports. This fact reinforces the usefulness of assessing the effect of training through the change in velocity against a certain absolute load or set of loads.

Another important application of this procedure is that it allows us to analyze the training effect on the entire force–velocity curve, and not only against the maximum load (which is seldom, if ever, encountered in competition in the vast majority of sports). We again stress that, in order to obtain the most correct and valid information, only the same absolute loads common to both pre- and post-training tests should be compared. Moreover, the maximum load measured in the initial test does not need to be equivalent to 1RM. Figure 3 shows a real example of assessing the effect of several weeks of training in the bench press exercise. This procedure allows us to assess the training effect against light, medium, and high loads. By contrast, if only the typical 1RM test were used to assess the training effect, we would only have information (which, by the way, is usually not very accurate) about the changes against the maximum load (i.e., in the zone of maximum load and minimum velocity of the force–velocity curve) and no information would be available on the training effect against light and medium loads (see Fig. 3 for more details).

As previously discussed, another relevant application of this procedure would be to know the evolution of the training effect throughout the training cycle [46, 50]. If, as training progresses, heavier absolute loads are lifted at the same velocity, the training effect will be positive, whereas the effect will be negative if the absolute loads lifted at that velocity are lower than previously. This application is essential because, by using this procedure, it would be possible to analyze the evolution of strength performance throughout the training period. Thus, for example, we could know when the greatest training effect has been achieved within a cycle, since peak performance does not necessarily occur at the end of the training cycle.

As can be observed, the applications derived from the analysis of the relationship between the training loads applied and the resulting neuromuscular effects are numerous and of great importance to improve our understanding of RT as well as to find out what are the most useful types of training protocols for the enhancement of athletic performance. Very valuable information can be derived from this type of analyses which had never been possible to date. Furthermore, it is important to consider that all this information is individually obtained for each athlete.

## Conclusions

This manuscript has exposed many errors and inconsistencies, both conceptual and terminological, that continue to exist in the field of RT. A particularly worrying and underlying problem is that of current terminology, which is often inappropriate, misleading and/or unnecessarily complex which fosters confusion and misconceptions, hindering the development of a sound and scientific-based training methodology. Unless these deficiencies are corrected, progress in our field will remain severely limited. Together with terminology, it seems necessary to modify (which in many cases involves clarifying and simplifying) many crucial aspects of the current approach, such as the very concept of maximal strength, the control and monitoring of the resistance exercise dose, the existing programming models, the training goals or objectives, and the evaluation of training effects. It thus seems evident that a paradigm shift is advisable to effectively address this situation. This new paradigm must guarantee a precise knowledge of the loads being applied, the effort they involve and their effects. To the best of our knowledge, currently this can only be achieved by monitoring movement velocity during training. However, a velocity-based resistance training approach does not guarantee that an RT program is effective for improving physical or athletic performance, since it does not prevent mistakes from being made when programming or prescribing training loads. The key contribution of this approach is that the monitoring of repetition velocity provides the necessary information to know the actual training loads that induce a specific effect on each athlete. This precise and individualized information has never been available before. The correct adoption of this revised paradigm will provide coaches and strength and conditioning professionals with accurate and objective information concerning the applied load (relative load, level of effort and training effect). This knowledge is essential to make rational and informed decisions and to improve the training methodology itself. In addition, the valuable information obtained by means of monitoring movement velocity may contribute to opening up new lines of research.

## Data Availability

Not applicable.

## Abbreviations

1RM: One-repetition maximum
BP: Block periodization
CMJ: Counter-movement jump
d: Distance
EI: Effort index
F: Force
m: Mass
NPT: Non-periodized training
*n*RM: Maximum number (*n*) of repetitions in a test or training set (e.g., 5RM, 10RM, etc.)
RFD: Rate of force development
RT: Resistance training
t: Time
T20: Time to cover 20 m running sprint
v: Velocity
V_1RM_: 1RM velocity
VL: Velocity loss
